# Frequency selective neuronal modulation triggers spreading depolarizations in the rat endothelin-1 model of stroke

**DOI:** 10.1177/0271678X211013656

**Published:** 2021-05-09

**Authors:** Paolo Bazzigaluppi, James Mester, Illsung L Joo, Iliya Weisspapir, Adrienne Dorr, Margaret M Koletar, Tina L Beckett, Houman Khosravani, Peter Carlen, Bojana Stefanovic

**Affiliations:** 1 Sunnybrook Research Institute, Physical Sciences, Toronto, ON, Canada; 2 Department of Medical Biophysics, Faculty of Medicine, University of Toronto, Toronto, ON, Canada; 3 Division of Neurology and Interdepartmental Division of Critical Care, Department of Medicine, Sunnybrook Health Sciences Centre, University of Toronto, Toronto, ON, Canada; 4 Interdepartmental Division of Critical Care, Department of Medicine, University of Toronto, Toronto, ON, Canada; 5 Krembil Research Institute, University of Toronto, Toronto, ON, Canada

**Keywords:** Focal cortical ischemia, optogenetic, peri infarct depolarizations, 2-photon laser scanning microscopy, local field potentials

## Abstract

Ischemia is one of the most common causes of acquired brain injury. Central to its noxious sequelae are spreading depolarizations (SDs), waves of persistent depolarizations which start at the location of the flow obstruction and expand outwards leading to excitotoxic damage. The majority of acute stage of stroke studies to date have focused on the phenomenology of SDs and their association with brain damage. In the current work, we investigated the role of peri-injection zone pyramidal neurons in triggering SDs by optogenetic stimulation in an endothelin-1 rat model of focal ischemia. Our concurrent two photon fluorescence microscopy data and local field potential recordings indicated that a ≥ 60% drop in cortical arteriolar red blood cell velocity was associated with SDs at the ET-1 injection site. SDs were also observed in the peri-injection zone, which subsequently exhibited elevated neuronal activity in the low-frequency bands. Critically, SDs were triggered by low- but not high-frequency optogenetic stimulation of peri-injection zone pyramidal neurons. Our findings depict a complex etiology of SDs post focal ischemia and reveal that effects of neuronal modulation exhibit spectral and spatial selectivity.

## Introduction

The most common causes of acquired brain injuries are stroke, traumatic brain injury, and hypoxic-ischaemic encephalopathy after cardiac arrest.^1,2^ Stroke in particular is in the top five causes of mortality^3^ and in survivors, permanent brain damage often leads to severe disability and loss of independence, making acquired brain injury a high-priority research focus area.^4^ Over the last fifty years, spreading depressions were observed in ischemic and hemorrhagic stroke, traumatic brain injury, and migraine.^5–7^ Recurrent spreading depolarizations (SDs) affect the progression of neurovascular damage following ischemia,^4,6^ and increase the degree and volume of ischemic lesion in a stepwise manner.^8–10^ In particular, spreading depolarizations lead to two types of neuronal activity depression: i) spreading depression of activity in mildly ischemic and isometabolic tissue, and ii) non-spreading depression of activity in severely ischemic tissue. Spreading depolarization may (or may not) induce spreading depression,^11^ but spreading depression necessarily co-occurs with spreading depolarization.^12^ The onset of the depolarization is associated with the breakdown of ion homeostasis, water influx (i.e. cytotoxic edema) and metabolic failure. These can be reversed -up to a point- by restoring perfusion.^13,14^ If the ischemia persists, however, the depolarization, cytotoxic edema and metabolic failure, which exhibits a wave-like behavior, with periods of exacerbations alternating with recovery phases.^12^ The simple and frequently invoked conceptual model posits the existence of an ischemic injection site, in which all neurons die rapidly, and a spatially distinct region of ischemic peri-injection zone of functionally challenged but surviving neurons. However, the pathological processes underlying neuronal injury -energetic failure, cytotoxic edema, and sustained depolarization- occur along a continuum in space and time.^4,15^ In particular, at least 15minutes of ischemia and persistent depolarization usually elapse before the first neurons, most proximal to the site of the vascular occlusion, die.^16–20^ Further depolarizations around this necrotic injection site lead to elevated metabolic demand and spatial expansion of the necrotic zone, in a centre-surround pattern of growth. The investigations of the pathophysiological mechanisms underlying SDs generation in different disease models yielded important insights into the biophysical origin of SDs(for review Dreier et al.^21^) and ushered the development of therapeutic approaches aimed at blocking the SDs.^22–24^

Recently, determinants of SDs have been re-examined. It has thus been reported that one way to trigger SDs in the mouse cortex is by mismatching cortical metabolic supply and demand via functional activation of the cortex^25^ so as to increase O_2_ demand in the acute phase following a 60-min occlusion of the distal middle cerebral artery. The authors concluded that neuronal activation during metabolic substrate shortage (due to reduced blood supply) triggered SDs. Previous studies used wide-field imaging to examine SDs occurrence in focal cortical ischemia and were able to visualize the SDs spread.^26,27^ The current study built up on these findings by analyzing the relationship between vRBC deficits in individual cortical microvessels and neuronal activity, in a model of focal cortical ischemia, the most common type of stroke.^28^ To this end, we used concurrent in situ two-photon fluorescence microscopy and intracerebral electrophysiology. As SDs are known to initiate in pyramidal neurons’ apical dendrites,^29–31^ we examined the role of pyramidal neurons in shaping the response to ischemia by inducing channelrhodopsin-2(ChR2) expression in them to allow frequency-band specific manipulation of the their activity. Our findings characterize the progression of neuronal dysfunction in response to cortical vRBC drop and shed light on the stimulation frequency selectivity of spreading depolarizations post focal ischemia.

## Methods

All experimental procedures followed the ARRIVE 2.0 guidelines and were approved by the Animal Care Committee of the Sunnybrook Research Institute, which adheres to the Policies and Guidelines of the Canadian Council on Animal Care and meets all the requirements of the Provincial Statute of Ontario, Animals for Research Act as well as those of the Canadian Federal Health of Animals Act. Nineteen adult male Sprague-Dawley rats (weight: 342 ± 46 g) were housed in pairs. All the data that support the findings of this study is presented.

### Viral transfection

Standard operating procedures for sterile surgery were employed. The AAV2.hSyn.ChR2(H134R)-eYFP.WPRE.hGH (#26973, Addgene USA) was diluted in PBS to a final concentration of 3.6 × 10^9^ vc/uL. Rats were anesthetized and maintained with isoflurane (5% for induction, 2.5% for maintenance), received saline (3 mL subcutaneously) for hydration, and Baytril antibiotic (5 mg/kg) and Xylocaine (7 mg/kg) local anesthetic applied around the surgical site. The rat was positioned on the stereotaxic frame, blood pressure monitored by pulse oximetry (MouseOx, STARR Life Sciences Corp, USA), and injection site body temperature maintained on a heating pad with rectal feedback probe (TC-1000 Temperature Controller, CWE Inc., USA). The head was shaved and skin prepared with antibacterial soap, isopropyl alcohol, and betadine iodine solution. A 3 cm midline incision was made from the eyes, then the skull was cleared of periosteal tissue. A burr hole was created (Micromotor drill, Stoelting Co., USA) at AP −4 mm, ML 2.5 mm, for viral injection using a Neurosyringe 1701 (#65460-05, HamiltonCompany, USA). One microlitre of the virus was administered at two cortical depths over 15minutes, at DV −1000 um and at DV −500 um, for a total of 7.2 × 10^9^ vc. The incision was cleaned and sutured with 4-0 Polysorb (Covidien #SL-5637), and the animal then placed in a pre-warmed recovery cage.

### Histopathology

Three animals were used for histopathological confirmation of viral distribution post transfections. Two weeks following viral transfections, animals were anesthetized with 5% isoflurane and transcardially perfused using PBS with 0.1% heparin, followed by 4% paraformaldehyde in PBS (PFA). Brains were collected and post fixed overnight in 4% PFA, followed by cryoprotection in 30% sucrose in PBS prior to sectioning. Forty µm free floating sections were stained with Guinea Pig anti-NeuN (1:500, Millipore ABN90) and Rabbit anti-GFAP (1:500, DAKO Z0334); secondary antibodies were used at 1:200 (Dk anti-GP 594, Jax Immuno Research #706-585-148 and Gt anti-Rb 647, Invitrogen # A21245) with NucBlue Fixed Cell Stain ReadyProbes reagent as per manufacturer’s instructions (2 drops/mL, DAPI; Molecular Probes by Life Technologies, # R37606). Images were collected using 10x and 20x magnification on a Zeiss Axio Observer Z1.

### Surgical preparation

Rats were induced under 5% isoflurane and then moved to a feedback-controlled temperature pad (CWE Inc, Ardmore, PA) where they were maintained at 37 °C under 2–2.5% isoflurane. The tail vein was cannulated for fluorophore injection. Throughout the experiments, systemic physiology was monitored with a pulse oximeter (MouseOx, STARR Life Sciences) for recording of breath rate, heart rate, arterial oxygen saturation, and pulse and breath distention. Rats were fixed on a stereotactic frame and their heads immobilized via incisor bar and ear bars. Ringer's lactate solution (130 mmol/L Na, 4 mmol/L K, 1.5 mmol/L Ca, 109 mmol/L Cl, 28 mmol/L lactate, 0.5–1mL volume, Hospira, Canada) and Xylocaine (10 mg/mL, 50–100 µL volume, AstraZeneca, Canada) were administered subcutaneously for hydration and local anesthesia, respectively. We used a dental drill to create a 6x3 mm craniotomy centered over the somatosensory cortex (centered at −3 mm AP, 3 mm ML). The skull cap and the dura were removed. For the simultaneous V_RBC_ and LFP recordings experiments, 1% agarose in phosphate-buffered saline (PBS, Sigma-Aldrich) was applied to the dura shortly prior to placement of a thin (<500µm) layer of transparent, silicone-based polydimethylsiloxane (PDMS^32^) over the craniotomy. The silicone was then secured with cyanoacrylate glue and an immersion well created using dental cement along the perimeter of the cranial window (Land Dental, USA). A 70 kDa Texas Red dextran (Invitrogen, USA) dissolved in PBS (8.33 mg/mL) was administered via the tail vein (25 mg/kg body weight).

### Two-photon fluorescence microscopy (2PFM)

**Figure 1.**
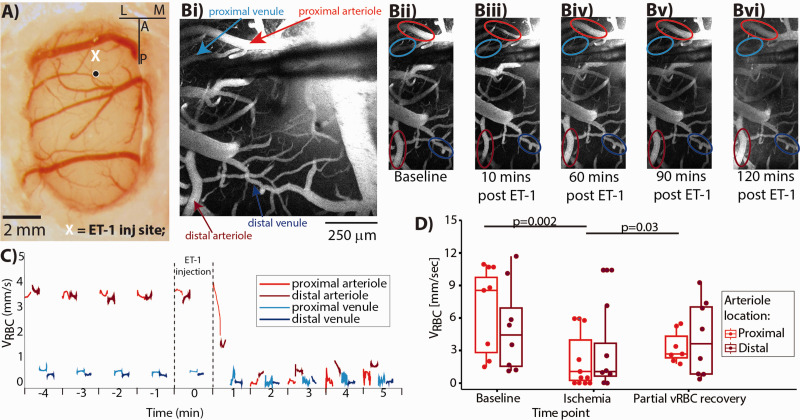
Simultaneous 2PLSM and LFP recordings. (a) Cerebral cortex covered with polydimethylsiloxane (PDMS), with the X marking the spot in the somatosensory area where the pipette was placed. (b) 2PFM images acquired at five timepoints: superimposed arrows (in Bi) and ellipses (Bii to Bvi) indicate the vessels in which line scans (for VRBC estimation) were acquired. Note that ET-1 elicits vasoconstriction of the proximal arteriole (red circle) but not of the proximal vein (cyan circle). (c) Time series of VRBC estimates during the first ten minutes of the experiment. The line scans acquisition sampled the vessels in an interleaved sequence. (d) Population VRBC data show significant reduction in VRBC during “ischemia” and “partial vRBC recovery” periods, when compared to that at “baseline.” The VRBC is still reduced two hours following ET-1 injection, while the vessels’ diameters recover within 1 hour of the ET-1 injection (between Biii and Biv), suggesting limited sensitivity of morphological scans for assessing cerebrovascular dynamics.

Rats were imaged on an FVMPE-RS multiphoton microscope (Olympus, Japan) using a 10x/0.6 NA/4 mmWD objective lens (Olympus, Japan). After positioning the rat under the microscope, the isoflurane was lowered to 1%. An Insight Ti:Sapphire laser (SpectraPhysics, USA) tuned to 900 nm was used to image Texas Red-labelled vasculature. A PMT and 485–540 nm barrier filter was used for signal detection. Line scanning (1.1–1.3 ms/line, 2µs/pixel) along the longitudinal axes of vessels was interleaved with raster scans of the whole field of view (1.2 × 1.2 mm). Due to the variations in the cortical cerebrovascular architecture and the constraints on the placement of the recording pipette, we were able to acquire line scans of 3 to 5 cortical penetrating vessels in each animal. We imaged at least one arteriole close to the ET-1 injection site (i.e. within 100 µm, from here on “proximal arteriole”), and in 5 out of 6 cases one arteriole far from the ET-1 injection site (i.e. farther than 1 mm, from here on “distal arteriole”). The breakdown of recorded vessels is reported in the Supplementary Table 1. Arterioles and venules were identified “on the fly” based on the direction of their RBC velocity in relation to the pial surface. Line scanning was conducted at baseline (5 to 10 minutes), during ET-1 injection (1 minute) and up to 120 minutes following ET-1 injection. In some instances, scanning had to be interrupted during the period following ET-1 injection to adjust the plane of focus and/or acquire structural scans (as in Figure 1(b)). Linescan analysis was adapted from previous work by Kim et al.^33^ and our group.^34^ Briefly, line-scanning particle image velocimetry (LS-PIV) allowed for recording of noisy data on fast moving red blood cells (RBCs). This method applies a cross-correlation to sequential linescans to capture RBC velocity (vRBC) providing accurate RBC speed estimation in higher-flow vessels.^33^

### Local field potential recordings

In the first set of experiments (simultaneous 2PFM and single-channel electrophysiology) local field potentials (LFPs) were acquired via a borosilicate glass micropipette (World Precision Instrument). The pipette was slid between the brim of the dental cement chamber built around the cranial window and the objective at −2 mm AP (anterior-posterior) and/-2.5 mm ML (medial-lateral), and then lowered into the cortex to approximately 150 µm below the brain surface. In the second set of experiments (electrophysiological recordings from two sites), two glass pipettes were lowered approximately 150 µm below the brain surface, the first at −2 mm AP and −2.5 mm ML; and the second 2 mm caudal from the first, at −4 mm AP and −2.5 mm. LFPs were amplified with an Axoclamp 700B (Axon Instruments) between DC and 1 kHz, sampled at 10 kHz with a DigiData1440 (Axon Instruments) and stored on a computer for offline analysis.

### ET-1 injections

ET-1 (Tocris) was dissolved on the day of the experiment in PBS (1 µg/1 µl) and injected through the most rostral glass pipette (which was also used for LFP recordings) using a pico-sptritzer (Picospritzer III, Science Products GmbH) over 1 minute. The ET-1 dose has been titrated to produce a targeted drop in vRBC; and the baseline vRBC under 1% isoflurane are well aligned with prior work under light isoflurane anesthesia or urethane anesthesia.^35,36^ Notwithstanding, the observed effects may be conservative estimates given that the isoflurane-induced anesthesia has been shown neuroprotective in the photothrombotic mouse model of stroke.^37^

### Optogenetic stimulation

To modulate neuronal activity, we stimulated neurons using a range of frequencies comprising each of the literature defined bands.^38^ To this end, we convolved sinusoidal waves at 1 Hz increments within the five bands of interest (theta: 2–10Hz, alpha: 10–15Hz, beta: 15-30Hz, low gamma: 30–80Hz; and high-gamma: 80-120Hz) in Matlab. Digital synthetic waveforms were then downsampled to 500 Hz, exported in the Arduino API at 12 bit resolution, and finally imported into an Arduino Due board (SAM3X8E Arm® Cortex®-M3 CPU) for D/A conversion. The resulting analog waveform was then fed to a 450 nm laser diode (Doric Lenses inc., Quebec, Canada) coupled to an optical fibre (200 µm diameter, 0.27 numerical aperture) that was positioned over the transfected area. Light intensity was set to ∼30 mW/mm^2^ as in our previous work.^39^

### Local field potential analysis

**Figure 2.**
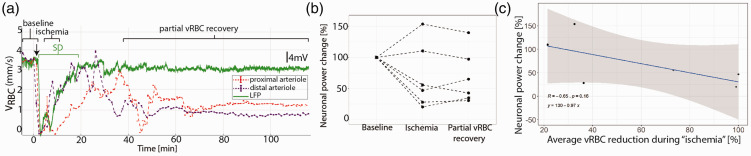
Neuronal activity following ET-1 injection. (a) Representative concomitant LFP (green) and VRBC (red in proximal-, purple in distal- arteriole) recordings showing that the ET-1 induced drop in VRBC is followed by a SD and that reperfusion occurs first in the vessels far from the ET-1 injection site. (b) Average neuronal power was reduced in the animals which exhibited a VRBC reduction to below 40% of the baseline level, while the two animals showing more modest perfusion decrease exhibited no reduction in neuronal power. (c) The average drop in VRBC between proximal and distal arterioles during “ischemia” is negatively correlated with the changes in neuronal power.

**Figure 3.**
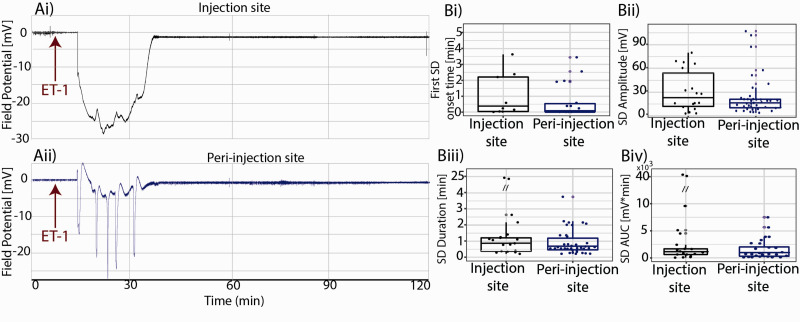
Injection site vs. peri-injection zone spontaneous SDs. SDs started within minutes and resolved within 56 minutes from ET-1 injection. ET-1 Injection site(Ai) showing the spreading depression followed by the SD, peri-injection zone(Aii) showing five SDs; population analysis showing SDs’ delay time(Bi), amplitude(Bii), duration(Biii) and AUC(Biv) recorded from the injection site and from the peri-injection zone.

Electrophysiological recordings were analyzed with in-house developed Matlab routines. Prior to power analysis, raw electrophysiological recordings were high-pass filtered at 1 Hz and line noise eliminated with a notch filter at 60 Hz. Peri-infarct depolarizations (SDs) were defined as negative deflections larger than 10 standard deviations over the baseline mean (of ∼4mV) that persisted for longer than 10 seconds. SDs were considered concluded once the LFP recovered to 80% of the baseline level, at which point a new baseline segment would begin. For Figure 2(b), neuronal power was estimated by computing the Fast Fourier Transform over two minutes of recording at baseline, during ischemia, and during the initial spontaneous reperfusion. For Figure 3(b) and the detection of Post-ischemic potentials (PIPs), we computed the band-wise continuous wavelet transform. PIPs were defined as periods when the coefficients of the continuous wavelet transform in the theta and alpha bands were two standard deviations above the mean of baseline for longer than 2 seconds.^40^

### Statistical analysis

Unless specified otherwise, linear mixed effect (lme) modelling was used in the statistical analysis as it recognizes the relationship between repeated observations on the same subjects while allowing for unbalanced groups (here present due to attrition). Lme thus produces robust and sensible restricted maximum likelihood estimates in the presence of unbalanced allocation of subjects by factor.^41^ SDs amplitude, duration and AUC, and PIPs counts were modelled as linear functions of group (spontaneous or optostimulation evoked), while subjects were treated as random effects, thus accounting for across subject variation. Normality was assessed by Shapiro-Wilk’s test (alpha 5%), results are expressed as mean ± S.D. unless specified otherwise.

## Results

**Figure 4. fig4-0271678X211013656:**
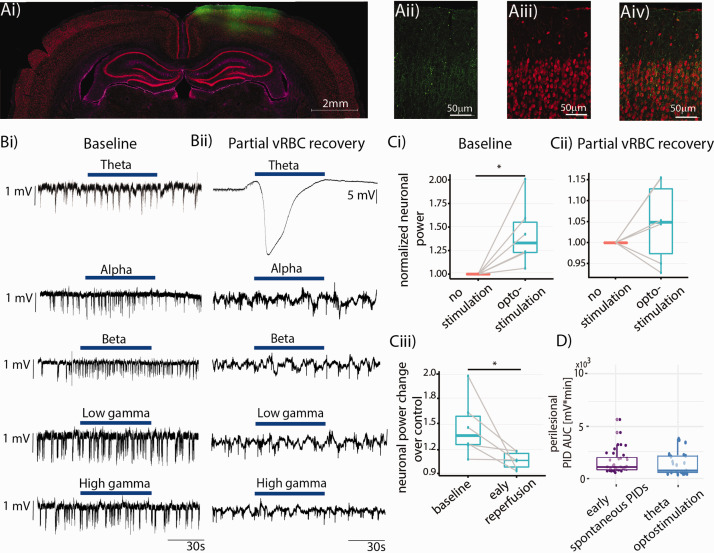
Optogenetic manipulation of the peri-injection zone. (a) Coronal section showing strong ChR2-YFP expression in the cortical area of interest, with layer II/III pyramidal neurons exhibiting green-yellow fluorescence (Aii: native YFP fluorescence from the reporter, Aiii: NeuN staining, Aiv: overlay, GFAP immunostaining presented in Supplementary Figure 4). (b) Representative LFP trace recorded from the peri-injection zone showing the effects of optogenetic stimulation (blue bar) at different bands at “baseline” (Bi) and during “partial vRBC recovery” (Bii). Note that theta band stimulation under physiological conditions elicits a neuronal response, but does not trigger SDs. (c) Population analysis showing the effects of optostimulation on neuronal power at “baseline” (Ci) and during “partial vRBC recovery” (Cii), and their comparison (Ciii); (d) population analysis indicated that the AUC of spontaneous vs. optogenetic stimulation induced SDs are commensurate.

Stroke elicits a cascade of aberrations in vascular function and neuronal activity both focally, at the site of flow decrement and in the surrounding brain tissue. In the first set of experiments, we characterized the drop in vRBC elicited by intracerebral ET-1 microinjection, and through concomitant electrophysiological recording, showed the profile of accompanying spreading depolarizations (SDs). Our findings on the hemodynamic deficit progression build upon the cortical RBC velocity data in stroke models to date.^35,42,43^ Subsequently, we examined the role of peri-lesional cortical layer 2/3 pyramidal neurons, pivotal players in cortical information processing,^44^ in the SDs generation. Higher background noise in electrophysiological traces of Figure 2 (*vs*. those of Figures 3 [Fig fig4-0271678X211013656]to 5) resulted from the interference caused by the microscopy equipment during concurrent electrophysiological recordings and 2PFM imaging.

### Endothelin 1 induced ischemic sequelae

Endothelin-1 is known to induce vasoconstriction followed by gradual reperfusion, recapitulating cerebrovascular dynamic post stroke in patients.^45^ ET-1 interacts with Endothelin-1A receptors, which are expressed preferentially in the arteriolar walls in the rat brain.^46–48^ Accordingly, we observed a vasoconstricting effect of ET-1 in arterioles, but not in venules (Figure 1(b)): the changes in on venular V_RBC_ were thus secondary to arteriolar V_RBC_ changes. Within two minutes of injecting ET-1, we observed a rapid arteriolar V_RBC_ drop, ranging from 46% to 100% in the arterioles close to the ET-1 injection site, and from 19 to 100% in the arterioles further away from the ET-1 injection site (cf. Figure 1(c) for a representative subject data; data from all subjects are displayed in Supplementary Table 2, Laser Doppler Flowmetry from the peri-injected cortex showed in Supplementary Figure 5). In five out of six of experiments, V_RBC_ transiently recovered to >80% of its baseline level, and then plateaued at ∼35% of the baseline (Figure 1(d)).

While the complete occlusion of the vessels lasted only minutes, red blood cell velocity remained attenuated in all ET-1injected animals throughout the 120-minute post-ET-1 observation period.

We segmented the post ET-1 period into three sub-periods: “baseline” (prior the ET-1 injection), “ischemia” (the five minutes following the peak drop in VRBC), and “partial vRBC recovery” (from the time VRBC reached 80% of baseline level to the end of the recording). Following ET-1 injection, the volley that marked the start of the “partial vRBC recovery” occurred 42 ± 12 minutes in proximal arterioles and 36 ± 17 minutes in distal arterioles. Linear Mixed Effects model revealed that on average, VRBC of proximal arterioles decreased by 54 ± 4%, from 6.0 ± 2.2 mm/s at “baseline” to 2.6 ± 1.8 mm/sec during “ischemia” (p = 0.002), and then during “partial vRBC recovery,” which started 45 ± 13minutes following ET-1 injection (Supplementary Table 2), VRBC increased to 4.1 ± 2.2 mm/sec, thus rising by 58 ± 8% from its level during ischemia (p = 0.03).

### V_RBC_ drop and SDs

While monitoring the V_RBC_, we also recorded Local Field Potentials at the ET-1 injection site so as to investigate the relationship between microvascular and neuronal function. Within ∼5minutes of the initial drop in V_RBC_ in the proximal arteriole, we invariably observed at least one peri-infarct depolarization wave (Figure 2(a), SDs). We observed a subject-wise mean of 1.6 ± 1.8 SDs at the ET-1 injection site (n = 11 SDs), with a mean amplitude of 9.5 ± 7.1 mV and a mean duration of 220 ± 178.2 sec, starting 284 ± 149.1 sec after ET-1 injection (two animals did not show halving of V_RBC_ post ET-1 injection and exhibited no SDs either). While the complete occlusion of the vessels lasted only minutes, red blood cell velocity remained attenuated in all ET-1injected animals throughout the 120-minute post-ET-1 observation period. Across animals, the SDs were generated when V_RBC_ fell below 35 ± 10% of the baseline level.

We anticipated that the degree and duration of V_RBC_ change would predict neuronal behavior, so we calculated the area under the product of the V_RBC_ and time during “ischemia”(i.e., average distance travelled by RBCs) and used it to predict change in neuronal power between “baseline” and “ischemia”(Figure 2(b), Supplementary Figure 1). We found that the change in neuronal power was inversely correlated with the regional decrease in distance travelled by RBCs (Figure 2(c), p = −0.12, r = 0.65). In light of the previously identified link between SDs and expansion of the peri-injection zone,^9,49^ we next investigated whether the degree of perfusion drop were the major determinant of SDs amplitude. To answer this question, we calculated the area under the product of the V_RBC_ and time during “ischemia”(therefore distance travelled by RBCs) and used it to predict the area under the product of SDs amplitude and time during “ischemia.”

#### Neurovascular changes at the injection site vs. in peri-injection zone

Simultaneous assessment of vRBC and LFP revealed a coupling between microvascular and neuronal functional changes at the ischemic injection site. In eleven animals, we recorded 39 SDs in the peri-injection zone and 18 SDs in the injection site, demonstrating a spatial heterogeneity in SDs’ origin (Chi Squared test: null-hypothesis: uniform probability between injection site and peri-injection zone, df = 1, p = 0.02). The time course of LFP recordings from a representative subject (Figure 3) shows that the SD led to the so-called negative ultraslow potential at the injection site (Ai), and peri-injection zone (Aii). These phenomena appeared to be caused by hypoxia (i.e. hypoxic depolarizations) but given the absence of independent assessment of tissue oxygenation we refrain from using that term. Irrespective of their location, SDs showed the same delay relative to the ET-1 injection time (Figure 3(Bi), median onset time(min)±C.I.: 0.91 ± 0.8 min vs. 0.98 ± 0.5 min, p-value: 0.64, Linear Mixed Effects Model with delay as a response variable, recording location as a fixed factor and subject as a random effect). SDs’ amplitude, duration and Area Under the Curve (AUC) were not different between the injection site and the peri-injection zone, in line with the notion of a spatial continuum in the SDs’ (Figure 3(Cii) and (Ciii), median amplitude(mV) ± C.I.: 22.1 ± 12.9 mV *vs.* 17.1 ± 8.1 mV, p-value: 0.46; median duration (min) ± C.I.: 0.96 ± 2.5 min *vs.* 0.58 ± 0.4 min, p-value: 0.14; median AUC(min*mV) ± C.I.: 1117  ±  4909 min*mV *vs.* 732 ± 1526 min*mV, p = 0.06; Linear Mixed Effects Model with amplitude or duration or AUC as response variable, recording location as a fixed factor and subject as a random effect). Furthermore, across all animals, SDs started within minutes and resolved within an hour of ET-1 injection (Figure 3(a)).

#### Optogenetic manipulation of the peri-injection zone

To gain a deeper understanding of the generative process of SDs, we next examined the cellular origins of SDs in the peri-injection zone. Since we did not observe any spontaneous SDs after the first hour following ischemia, we examined the susceptibility of peri-injection zone to exogenously induced SDs in the second hour post ET-1 injection. To that end, we transfected the rat cortices with Channelrhodopsin2 so as to enable photostimulation-based modulation of the activity of pyramidal neurons in the peri-injection zone. Figure 4 shows a coronal slice of a rat brain transfected with the AAV2.hSyn.ChR2(H134R)-eYFP.WPRE.hGH at −4.5 mm AP, −2.5 mm ML and −1 mm DV(dorso-ventral) seventeen days preceding the ET-1 injection. ChR2 expression distribution is assessed by YFP fluorescence, observed in pyramidal neurons’ somas and dendrites in a ∼1 mm radius around the transfection site (Figure 4(Aii), (Aiii) and (Aiv)). We hypothesized that pyramidal neurons will respond to photostimulation differently under metabolically challenged conditions *vs*. under physiological conditions, and that this difference would become particularly salient upon stimulation in the theta band, i.e. in resonance with their spontaneous firing.^44,50^

On the experiment day, we placed two glass pipettes for LFP recordings, one at the ET-1 “injection site”, and the second ∼2 mm caudally, so as to record neuronal activity from the peri-injection zone which coincided with the area transfected with ChR2 (from here on, “peri-injection zone”). Careful placement of the pipettes is of critical importance to avoid major damage to parenchyma and CSD triggering, as described in our previous studies.^51–54^ Before ET-1 injection, we illuminated the cortex with 450 nm light at different frequency bands (theta, alpha, beta, low gamma, and high gamma) for 60 s, with 60-s interstimulus interval, and with stimulus frequency bands presented in random order. Figure 4(Bi) and (Bii) show representative optogenetic stimulation at different bands at “baseline” and during “partial vRBC recovery,” respectively. Optostimulation of the peri-injection zone at “baseline” resulted in a 42.5 ± 7.2% increase in neuronal power (Figure 4(Ci), paired t-test, one tail, p = 0.014, N = 6), while the same stimulation administered during “partial vRBC recovery” did not elicit a significant increase in neuronal power (Figure 4(Cii), difference between groups: 4.7 ± 3.1%, paired t-test, one tail, N = 6, p = 0.143). We then compared the neuronal response to stimulation at the two timepoints and observed that neuronal response to optostimulation during “partial vRBC recovery” is significantly smaller than that to the same stimulation at “baseline” (effect size: 18.9 ± 2.1%, paired t-test, one tail, N = 6, p = 0.03). Importantly, while optostimulation in alpha, beta, low gamma and high gamma bands during “partial vRBC recovery” barely elicited a neuronal response, optostimulation in theta band resulted in SDs in 80% of our experiments (full dataset from individual subjects in the Supplementary Figure 2). These data showed that optogenetic stimulation at the resonant frequency of pyramidal neurons had a significantly higher probability of triggering SDs than did the optogenetic stimulation in the other bands (Chi Squared test, followed by pairwise comparison with FDR correction: null-hypothesis: uniform probability between bands, df = 4, p = 2.2e-16).

We further hypothesized that the trigger (spontaneous *vs*. theta band optostimulation) would determine the size of the SDs. Linear model of peri-injection zonel SDs’ AUC with stimulation type as fixed effects and subject as random effect revealed that spontaneous and optostimulation evoked SDs were indistinguishable (Figure 4(d), median AUC(min*mV)±95% C.I.: 732.6 ± 1526 vs. 448 ± 443, p = 0.4, Figure 4(d)). This finding indicated that theta optostimulation can be reliably used to trigger SDs comparable to the ones occurring spontaneously and therefore may be useful for testing novel therapies for curbing SDs.

#### Post-ischemic potentials

**Figure 5.**
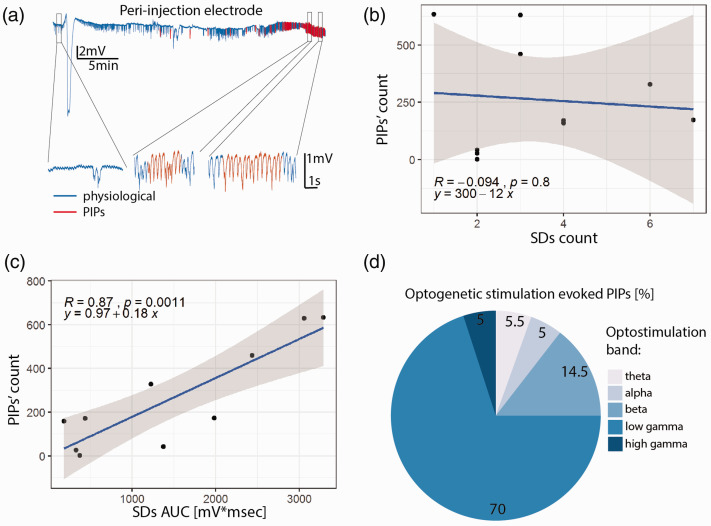
Post-ischemic potentials (PIPs). (a) Representative LFP recording from the peri-injection zone (blue trace) with segments identified as PIPs rendered in red. Insets show segments of the recording at baseline and during PIPs. The PIP count is not correlated with the SD count (b), but is strongly correlated with SDs AUC (c). (d) Photostimulation in the beta and low gamma bands accounted for ∼85% of the photostimulation-induced PIPs.

Over the last few decades, a number of studies have reported abnormal electrophysiological findings in the patients in the hours following ischemia.^55^ To characterize our model, we followed a well-established classification method^56–59^ that divides neuronal activity into a high amplitude slow (HAS) activity (oscillation frequency below the alpha range with a high amplitude at seizure onset) and a low amplitude fast (LAF) activity (oscillations in the beta to gamma range). Whereas we did not detect any LAF, we did observe HAS activity in the peri-injection zone, following the last SD and lasting until the end of the recordings (Figure 5(a)). We have termed the abnormal neuronal activity recorded in our experiments post-ischemic potentials (PIP) in light of their manifesting concomitantly with reduced cortical perfusion. These spontaneous PIPs lasted on average 2.3 ± 0.5s.

In light of the co-occurrence of SDs and seizures in the clinic^60,61^ and our observation that SDs precede PIPs, we investigated whether the occurrence of spontaneous PIPs depends on the number of SDs or their AUC. While the number of SDs did not predict PIP load (Figure 4(b), p = 0.8, r = −0.1), SDs’ AUC was a strong predictor of PIP number (Figure 5(c), p = 0.001, r = 0.9). Since the AUC -unlike the simple count- incorporates the duration of the SDs, and SDs are likely caused by insufficient metabolic support of neuronal activity (our data and von Bornstadt et al.^25^) this observation indicates that the probability of PIPs increases with lengthening of the period spent in a metabolically challenged state.

We then hypothesized that pyramidal neuronal dysfunction underlies PIPs, i.e. that pyramidal activation would be central for the generation of PIPs. As for SDs, the probability to trigger PIPs by optostimulation depended on the frequency-band of the optostimulation (subject-wise data set presented in Supplementary Figure 3). Chi-Squared test revealed a significant difference between the observed probabilities of PIPs occurring during photostimulation in different bands (null-hypothesis: uniform probability across bands, df = 4, p = 2.2e-16). Pairwise comparisons with false discovery rate correction revealed that photostimulation in the low gamma and beta bands was significantly more likely to trigger PIPs than the ones in the other three bands (p = 2.2e-16 for low gamma, p < 0.005 for beta, Figure 5(d)). Taken together, these data indicate that optogenetic stimulation of pyramidal neurons in the low frequency band (theta) triggers SDs whereas higher frequency band (beta and gamma) photostimulation elicits PIPs.

## Discussion

The present study identified the threshold of cerebrovascular dysfunction necessary to trigger SDs in a model of focal cortical ischemia, defined the role of pyramidal neurons in generating SDs, and revealed that a unique pattern of neuronal dysfunction in the peri-injection zone arises during spontaneous reperfusion: we termed these abnormal activity patterns, perfusion changes related potentials (PIPs). The high spatial selectivity and cellular specificity of Channelrhodopsin-2 transfection allowed us to modulate neuronal activity and trigger SDs with high fidelity following photostimulation at the resonant frequency of pyramidal neurons, thereby revealing that SDs are triggered by the activation of pyramidal neurons at low frequencies, whereas higher frequency stimulation of pyramids elicits PIPs. Our findings, in a model of focal cortical ischemia, build upon previous work on large-vessel stroke.^25,62^

### Endogenous biomarkers of adverse outcomes

Our experiments showed that following focal cortical ischemia: *i*) SDs appear in the ET-1 injection site and in the peri-injection zone following a drop in local vRBC above 60%; *ii*) spontaneous SDs and PIPs present in a robust spatio-temporal pattern, with SDs manifesting at the ET-1 injection site and in the peri-injection zone, and PIPs appearing exclusively in the peri-injection zone and strictly following SDs. The threshold of 60% drop in V_RBC_ necessary to trigger SDs observed here is well in line with the 70% threshold observed in the MCAO model,^62,63^ and to the 45% observed by Bere and collaborators.^64^ In both cases, however, a drop in vRBC by 60–70% may be a conservative estimate as the isoflurane anesthesia used in those studies is vasodilatory and known to reduce the volume of vascular bed affected by ischemia, as demonstrated in the photothrombotic model of stroke.^37^

Notably, Continuous Arterial Spin Labelling Magnetic Resonance Imaging (CASL-MRI) has been used extensively by us and others to investigate cerebrovascular responses to ischemia on the mesoscopic scale. However, mapping the vRBC responses to SD is technically particularly challenging due to the requirement for simultaneous intracortical electrophysiological recordings and CASL-MRI and low SNR of ASL. To characterize the changes in the hemodynamics more comprehensively, future work should incorporate novel 3D scanning approaches to estimate vessel-wise caliber changes in addition to RBC velocities. SDs induce excitotoxic damage, and thereby expand the necrotic injection site^7,12,65^; and clusters of SDs predict poor functional recovery.^66^ On the other hand, it is of note that SDs’ invasion of regions distant from the ischemic injection site may induce protective preconditioning,^67,68^ adaptive synaptic plasticity,^68^ and neurogenesis,^69^ therefore being beneficial for functional recovery.^9,12^

Following partial vRBC recovery, we observed sustained increase in neuronal activity in the lower frequency bands, which we termed post-ischemic potentials. Anomalous neuronal activity has been observed in the hours to days following ischemia, hemorrhagic stroke and traumatic brain injury and in some instances it has been referred to as seizure-like events.^4^ The electrophysiological traces recorded in this study, however, did not exhibit the large initial depolarization characteristic of seizure-like event, so we opted for a different nomenclature. The putative relation between PIPs and seizure-like events, and consequences of elevated theta and alpha band neuronal power, i.e. PIPs, for functional outcome from stroke require further investigation.

### Role of pyramidal neurons in SDs and PIPs

Our data demonstrate that the stimulation of pyramidal neurons at low frequencies triggers SDs, while their stimulation in beta and low gamma preferentially triggers PIPs. While SDs are common to other stroke models, to the best of our knowledge, our work constitutes the first report of PIPs. The functional consequences of PIP, if any, are outside the scope of this study and require further investigation. Of note, because ChR2 with H134R mutation has a channel closing time constant just below 18 ms, ChR2(H134R) does respond, resulting in distinct action potentials, to photostimulation up to about 60 Hz (Lin 2011), but responses to stimulation above 60 Hz cannot be readily interpreted. The segregation of frequencies for the optostimulation was chosen to maximize its effects on pyramidal neurons: *in vivo* recordings show that pyramidal cells spontaneously fire between 5 and 20 Hz in awake resting state^70–72^ and over 100 Hz in response to sensory stimuli.^73^ Our results suggest that SDs may also be triggered at rest, when neuronal networks are synchronized, by the entrainment induced by optogenetic activation.

### Ischemic model considerations

In the current work, we examined the neurovascular sequelae following focal cortical ischemia induced by ET-1 so as to gain deeper understanding of the origin of neuronal dysfunction thus elicited. Previous studies^25,74^ on the origin of SDs relied on the occlusion of large vessels feeding the brain. While these models allow for precise control of occlusion duration and reperfusion timing, large vessel obstructions cause a widespread cortical and subcortical drop in perfusion, inducing neurovascular damage across extensive swaths of tissue.^45,75^ Conversely, the ET-1 model allows for spatial targeting of the ischemia and, given the gradual reperfusion following the brief complete vessel constriction (over the course of 20 hours in the rat^76^) the creation of a necrotic injection site surrounded by a functionally-challenged but salvageable peri-ischemic tissue, thereby mimicking the vRBC dynamics observed in patients and yielding a significant perilesional volume.^75^ In the first two hours following ET-1 injection, we here observed an early spontaneous recovery in red blood cell velocities starting ∼45mins post ET-1 injection and stabilizing at ∼65% of the baseline level over the following hour, well in line with a 0.5 to 0.7 peri-to-contralesional lateralization in cortical perfusion observed on perfusion MRI one hour post ET-1 injection.^76^ A significant limitation of the current work is that the assessment of the hemodynamic impairment was restricted to red blood cell velocity, whereas the oxygen transport from the microvasculature into tissue is determined by convective red blood cell flux, thus being influenced by hematocrit in addition to red blood cell velocity.^77^ Further technological developments are thus needed to enable high spatial and temporal resolution hematocrit mapping. Of note, given the spatial gradient of the ET-1 injection on the surrounding vessels, the integration of the signal across many vessels that underlie contrast generation in Doppler sonography (as well as MRI and PET) is expected to result in the average signal attenuation being smaller that the drop observed in individual arterioles proximal to the injection site. The difference results from varying spatial distributions of different ET-1 receptors in the brain. While ET-A receptors are the major receptor subtype in the cerebral vasculature and neurons,^78^ astrocytes predominantly express the ET-B receptor subtype.^79,80^ Furthermore, ET-1 suppresses endothelial nitric oxide production,^81^ and promotes Na^+^ influx/K^+^ efflux from brain capillaries,^82^ which could contribute to the generation and propagation of SDs.

### From bench to bedside

The presently used focal ischemic stroke model destroys 5-7% of the adult rat forebrain tissue,^54^ thereby recapitulating well the 5.3% (54 mL) volume of the forebrain tissue damaged by the typical supratentorial infarct volume in the patients.^83^ The preclinical model that we employed allowed us to simultaneously investigate vascular and neuronal sequelae at the injection site and in the peri-injection zone. Thanks to these measurements, we were able to associate vascular and neuronal phenomena, concluding that SDs were generated only during the initial phase of stroke, when V_RBC_ fell to below 40% of its pre-stroke level. While this number may not be directly translatable to humans due to differences in collateral perfusion between species and the use of anesthetics in our experiment, our and others’ work indicate that resting perfusion is significantly higher than the level necessary for supporting resting brain activity,^62^ in support of such large perfusion deficit threshold. The high SDs’ spatial variability observed in this preclinical study and their disappearance following spontaneous reperfusion, undersinjection sites the importance of measuring neuronal state in individual subjects. This observation might also inform the design of next generation of stroke-response protocols, as MRI/CT scanning is expensive and often hard to access rapidly, next-generation of portable EEG headsets may be a viable alternative to monitor the pathological sequelae of cerebrovascular occlusion.^84,85^ On the whole, our findings suggest that early, in-the-ambulance EEG assessment of neuronal activity may be invaluable for estimating stroke progression stage and timing the appropriate treatment protocol. In particular, once timely and comprehensive quantitative assessments of neurovascular state become clinically available, the anticonvulsants and channel blockers of theta firing in pyramidal neurons may prove more effective treatments for focal stroke than they have been shown to date. Of note, however, SDs in stroke patients have been reported during sedation via high doses of propofol and midazolam – which though anticonvulsant seem not to have (strong enough) inhibitory effects on SDs.^86^

## Supplemental Material

sj-pdf-1-jcb-10.1177_0271678X211013656 - Supplemental material for Frequency selective neuronal modulation triggers spreading depolarizations in the rat endothelin-1 model of stroke Click here for additional data file. Supplemental material, sj-pdf-1-jcb-10.1177_0271678X211013656 for Frequency selective neuronal modulation triggers spreading depolarizations in the rat endothelin-1 model of stroke by Paolo Bazzigaluppi, James Mester, Illsung L Joo, Iliya Weisspapir, Adrienne Dorr, Margaret M Koletar, Tina L Beckett, Houman Khosravani, Peter Carlen and Bojana Stefanovic in Journal of Cerebral Blood Flow & Metabolism
