# Supplementary material for: Frequency selective neuronal modulation triggers spreading depolarizations in the rat endothelin-1 model of stroke
Source: J Cereb Blood Flow Metab. 2021 May 9;41(10):2756–68. doi: 10.1177/0271678X211013656 (PMC8504421; doi:10.1177/0271678X211013656)
Supplement: sj-pdf-1-jcb-10.1177_0271678X211013656 - Supplemental material for Frequency selective neuronal modulation triggers spreading depolarizations in the rat endothelin-1 model of stroke [file sj-pdf-1-jcb-10.1177_0271678X211013656.pdf]

Supplementary Material - *Optogenetic induction of peri-infarct spreading depolarization in the ET-1 rat model of focal cortical ischemia* (Bazzigaluppi, Mester, Joo, Weisspapir, Dorr, Koletar, Beckett, Khosravani, Carlen and Stefanovic)

Supplementary table 1. Vessels Scanned per animal

Subject_ID	Number of scanned vessel	Arterioles	Venules
1	5	3	2
2	3	2	1
3	4	2	2
4	5	3	2
5	5	3	2
6	4	2	2

Supplementary table 2. Vessel-wise V_{RBC} analysis

Subject Number	Vessel Type	Drop in Vrbc[%]	Time of reperfusion [min]	Average Vrbc after reperfusion [%]
1	proximal arteriole	-70.43	35	-51.23
1	distal arteriole	-76.04	21	-35.88
1	proximal venule	-63.75	51	-0.91
1	distal venule	-95.32	30	-34.82
1	proximal arteriole	-94.35	32	-37.68
2	proximal arteriole	-74.63	52	-75.86
2	proximal venule	-98.16	54	-100
2	distal arteriole	1.94	15	-27.27
3	distal arteriole	-100	NaN	NaN
3	distal venule	-96.92	NaN	NaN
3	proximal arteriole	-100	NaN	NaN
3	proximal venule	-93.31	NaN	NaN
4	proximal arteriole	-45.73	57	-76.26
4	proximal venule	-46.59	86	-43.61
4	distal arteriole	-51.36	76	-77.92
4	distal venule	-100	59	-73.37
4	distal arteriole	-19.25	77	-29.90
5	proximal arteriole	-45.95	40	-50.85
5	distal arteriole	-38.95	14	-40.92
5	distal venule	-31.88	33	-44.55
5	proximal venule	-40.80	48	-76.31
5	distal arteriole	2.95	14	-8.43

6	proximal arteriole	-98.87	27	-76.52
6	distal venule	-100	36	-53.23
6	distal arteriole	-99.35	33	-6.74
6	proximal venule	-98.15	18	63.79

Supplementary Figure 1:

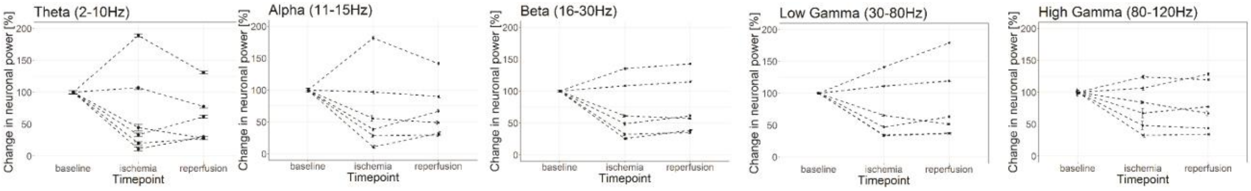

Supplementary Figure 1: Band-wise progression of neuronal power changes as “baseline”, “ischemia” and “early spontaneous reperfusion”

Supplementary Figure 2:

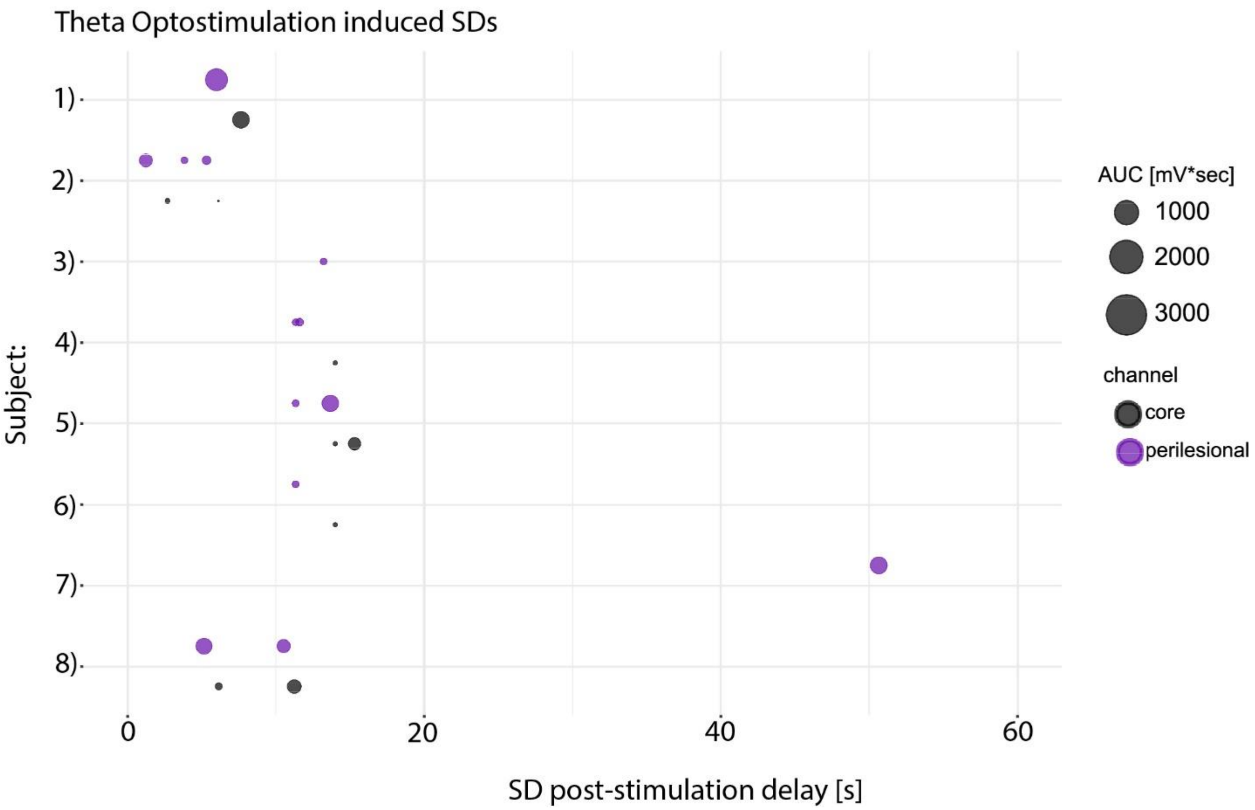

Supplementary Figure 2: Optostimulation induced SDs

Supplementary Figure 3:

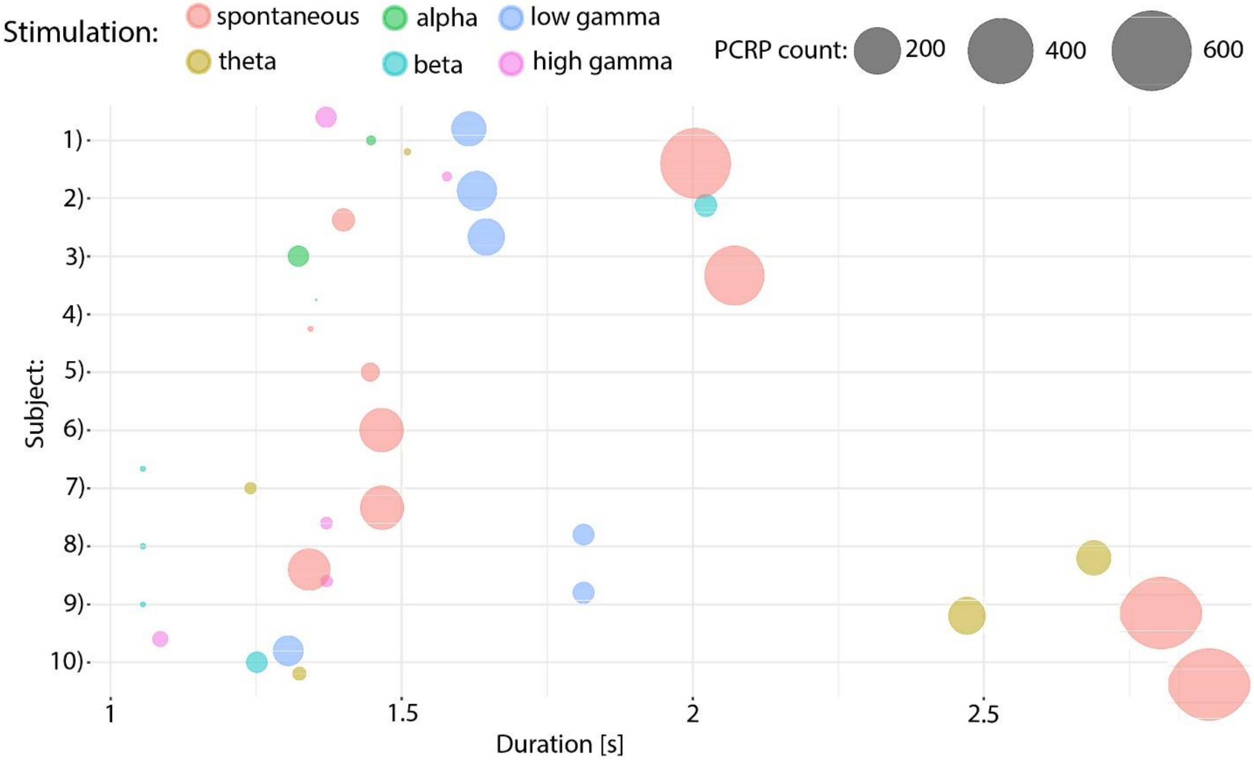

Supplementary Figure 3: Spontaneous PCRPs

Supplementary Figure 4:

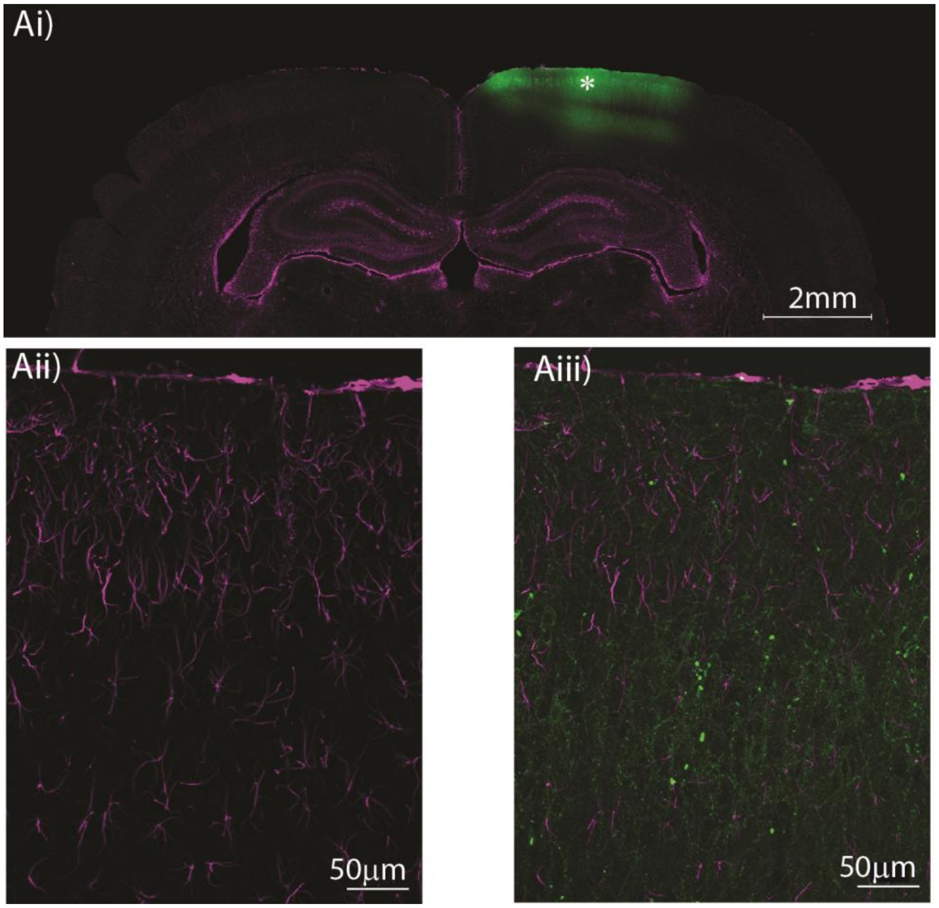

Supplementary Figure 4: Transfection does not cause astrogliosis. Ai) Coronal slice of representative rat transfected with AAV2.hSyn.ChR2(H134R)-eYFP.WPRE.hGH (#26973, Addgene USA). GFAP in purple and eYFP in green. The area at the asterisk is enlarged in Aii (GFAP only) and Aiii (GFAP and eYFP).

Supplementary Figure 5:

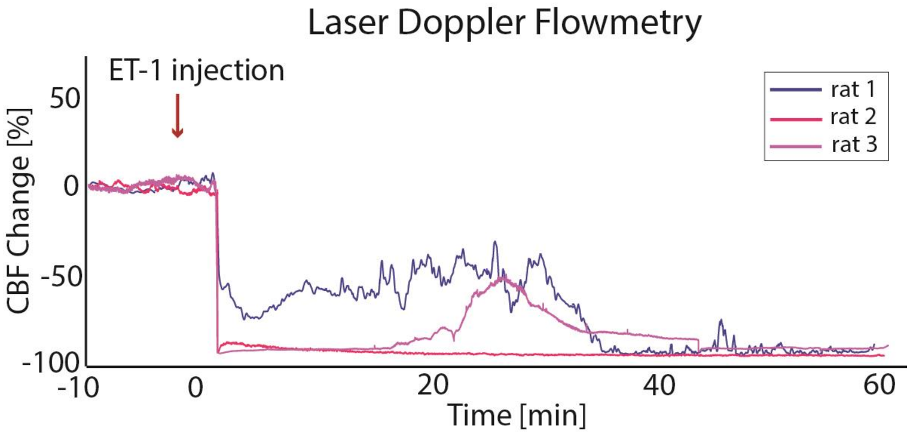

Supplementary Figure 5: A separate group of three male rats was dedicated to the study of cortical CBF from the peri-injected area by Laser Doppler Flowmetry (LDF100C, Biopac).
